# Supplementary material for: PPP2R1A regulates migration persistence through the NHSL1-containing WAVE Shell Complex
Source: Nat Commun. 2023 Jun 15;14:3541. doi: 10.1038/s41467-023-39276-w (PMC10272187; doi:10.1038/s41467-023-39276-w)
Supplement: Supplementary file 1 — Supplementary Information [file 41467_2023_39276_MOESM1_ESM.pdf]

SUPPLEMENTARY INFORMATION

Supplementary Figures ………………...… 2

Supplementary Methods ……………...…. 11

Supplementary References ………………. 13

Legends to Supplementary Tables ………. 14

SUPPLEMENTARY FIGURES

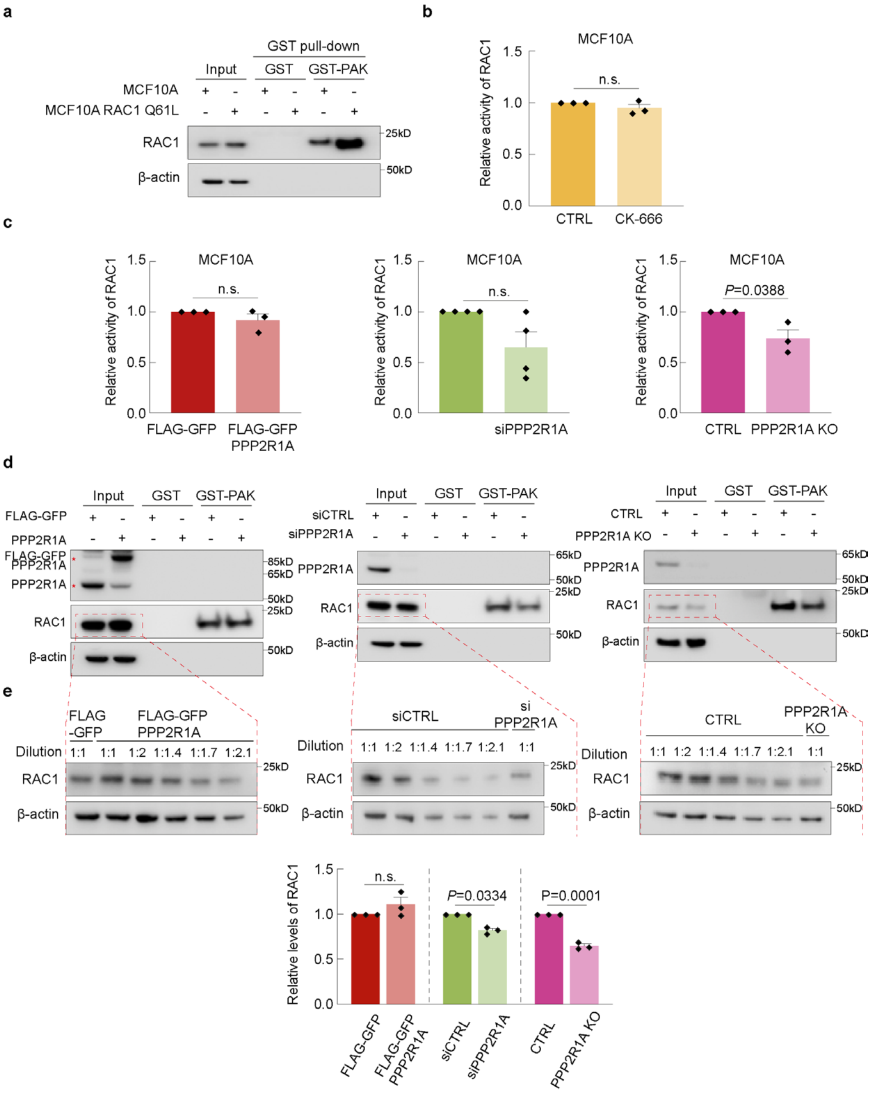

Supplementary Figure 1. Levels and activation of RAC1 in various conditions. (a) RAC1 activation by the GST-PAK pull-down assay in the genome edited MCF10A cell line that carries one RAC1 Q61L allele. 2 biological repeats with similar results. **(b)** Measurements of RAC1 activity by GLISA in MCF10A cells treated or not with 100 µM CK-666 for 16 h. 3 biological repeats, mean ± sem are plotted. **(c)** Measurements of RAC1 activity by ELISA in cells overexpressing or depleted for PPP2R1A. 3 or 4 biological repeats, mean ± sem are plotted. **(d)** Measurements of RAC1 activity and levels in cells overexpressing or depleted for PPP2R1A using the GST-PAK pull-down assay and RAC1 Western blots. 3 biological repeats with similar results. **(e)** Comparisons of RAC1 levels in different conditions using serial dilutions of the extract displaying more RAC1 by a factor of 1.2. Relative levels of RAC1 based on 3 independent Western blots. Student's t-test. Data are presented as mean values ± SEM. Statistical significance was calculated with two-tailed unpaired t-test (b,e) or Mann-Whitney test (c) and P values are indicated. n.s.: not significant. Source data are provided as a Source Data file.

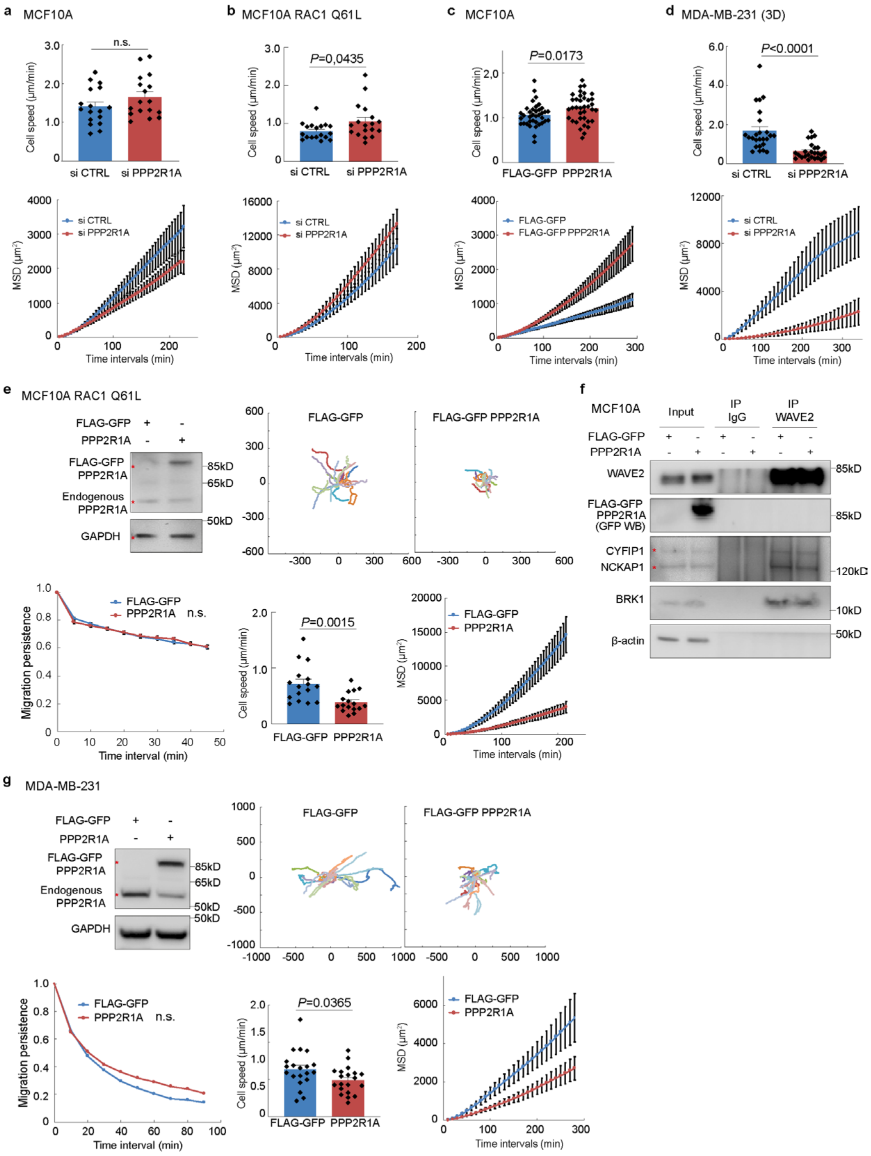

Supplementary Figure 2. Migration parameters of MCF10A and MDA-MB-231 cells. (a-d) Cell speed and MSD extracted from cell trajectories displayed in corresponding panels of figure 1. 3 biological repeats of the same experiment with similar results, only one is displayed. **(e)** Trajectories and migration parameters from random migration of single MCF10A RAC1 Q61L cells stably expressing FLAG-GFP or FLAG-GFP PPP2R1A. Tracking 7.5 h, n=16 cells. 2 biological repeats with similar results, only one is displayed. **(f)** Similar levels of WAVE2 and WRC in MCF10A cells stably expressing FLAG-GFP or FLAG-GFP PPP2R1A. 2 biological repeats with similar results. **(g)** MDA-MB-231 cell lines stably transfected with plasmids expressing FLAG-GFP or FLAG-GFP PPP2R1A were analyzed by Western blots with PPP2R1A or GAPDH antibodies. Cell trajectories, migration persistence, speed and MSD extracted from migration of single MDA-MB-231 cells embedded in 3D collagen type I gels. Tracking 10 h, n=20 cells. 3 biological repeats with similar results, only one is displayed. Data are presented as mean ± SEM. Statistical significance was calculated with two-tailed unpaired t-test and P values are indicated. Source data are provided as a Source Data file. n.s.: not significant.

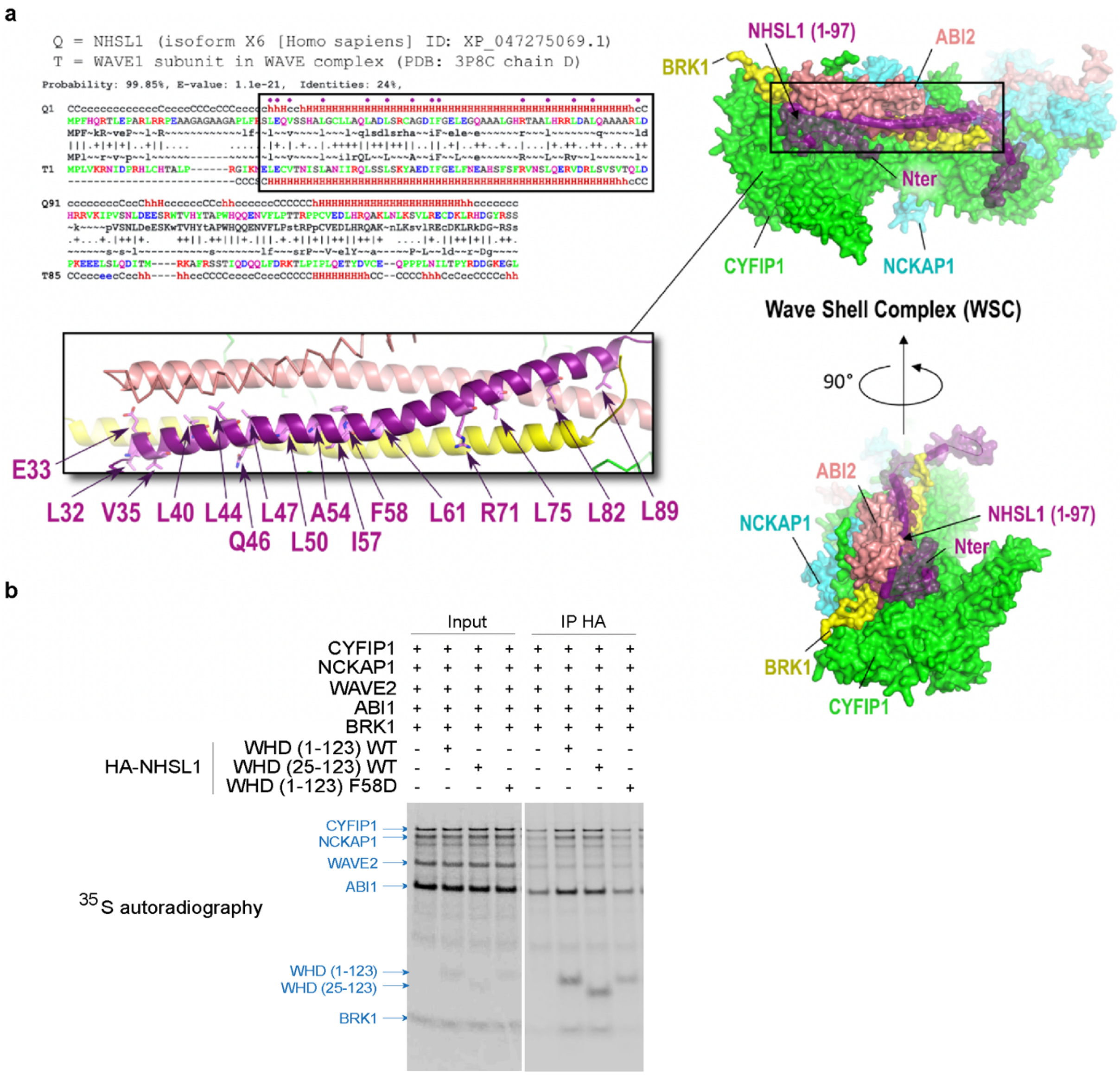

Supplementary Figure 3. The WSC is organized along a heterotrimeric coiled coil. (a) Structural model of the WSC generated by AlphaFold2. Sequence alignment between the profile of NHSL1 (line Q, 1-160 region) and that of the WAVE1 subunit (line T) extracted from the structure of the WAVE Regulatory Complex (PDB:3P8C [https://www.wwpdb.org/pdb?id=pdb_00003p8c]). The purple dots above the query sequence highlight the identical residue positions between NHSL1 and WAVE1. The identical residues are shown as sticks in the heterotrimeric coiled-coil region of NHSL1, BRK1 and ABI2. The structural model displayed with surface representation has been obtained with full-length CYFIP1, NCKAP1, BRK1, NHSL1 (1-123) and ABI2 (1-160). **(b)** *In vitro* translation of plasmids encoding subunits encoding the WRC and HA tagged fragments of the NHSL1 WHD. The first 25 amino-acids are dispensable to assemble a WSC, but the F58D mutation reduces WSC assembly to background levels. This experiment has been performed once.

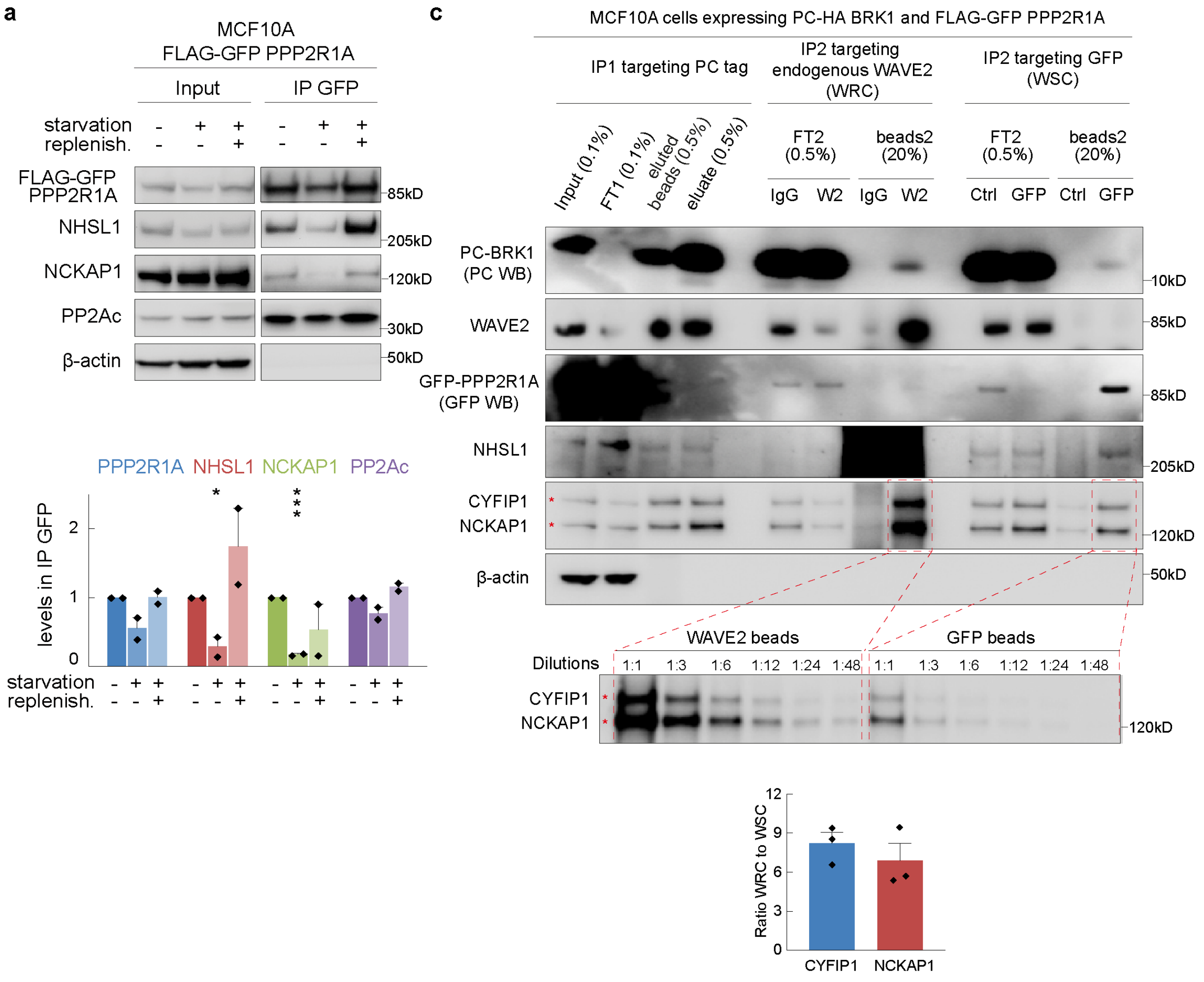

Supplementary Figure 4. Levels of WSC. (a) Down-regulation of the WSC by starvation. MCF10A cells were depleted of serum and EGF for 36 h and the medium was replenished for 16 h. Western blot and quantification of 3 independent experiments by densitometry. Data are presented as mean ± SEM. Statistical significance was calculated with two-tailed unpaired t-test and P values are indicated. **(b)** Relative amounts of WSC and WRC. A lysate prepared from the stable cell line expressing PC-HA-Brk1 and FLAG-GFP-PPP2R1A was subjected to two sequential native immunoprecipitations (IPs). The complexes containing PC-HA-Brk1 were selected by a first IP trough the PC mAb and eluted by Ca^{2+} chelation. The PC eluate was then the starting material for a second IP, either using WAVE2 Ab or using GFP trap beads to select the WRC or the WSC, respectively. The second IPs were quantitative as they depleted the lysate of the respective complex. The amounts of WRC and WSC captured on beads of the second IP were compared by serial dilutions. There is approximately 7 to 8 times less WSC than WRC in this cell line. This is probably an overestimation of the WSC given that FLAG-GFP is overexpressed and that WAVE2 is less completely depleted by the 2nd IP than GFP. FT: Flow through. Source data are provided as a Source Data file.

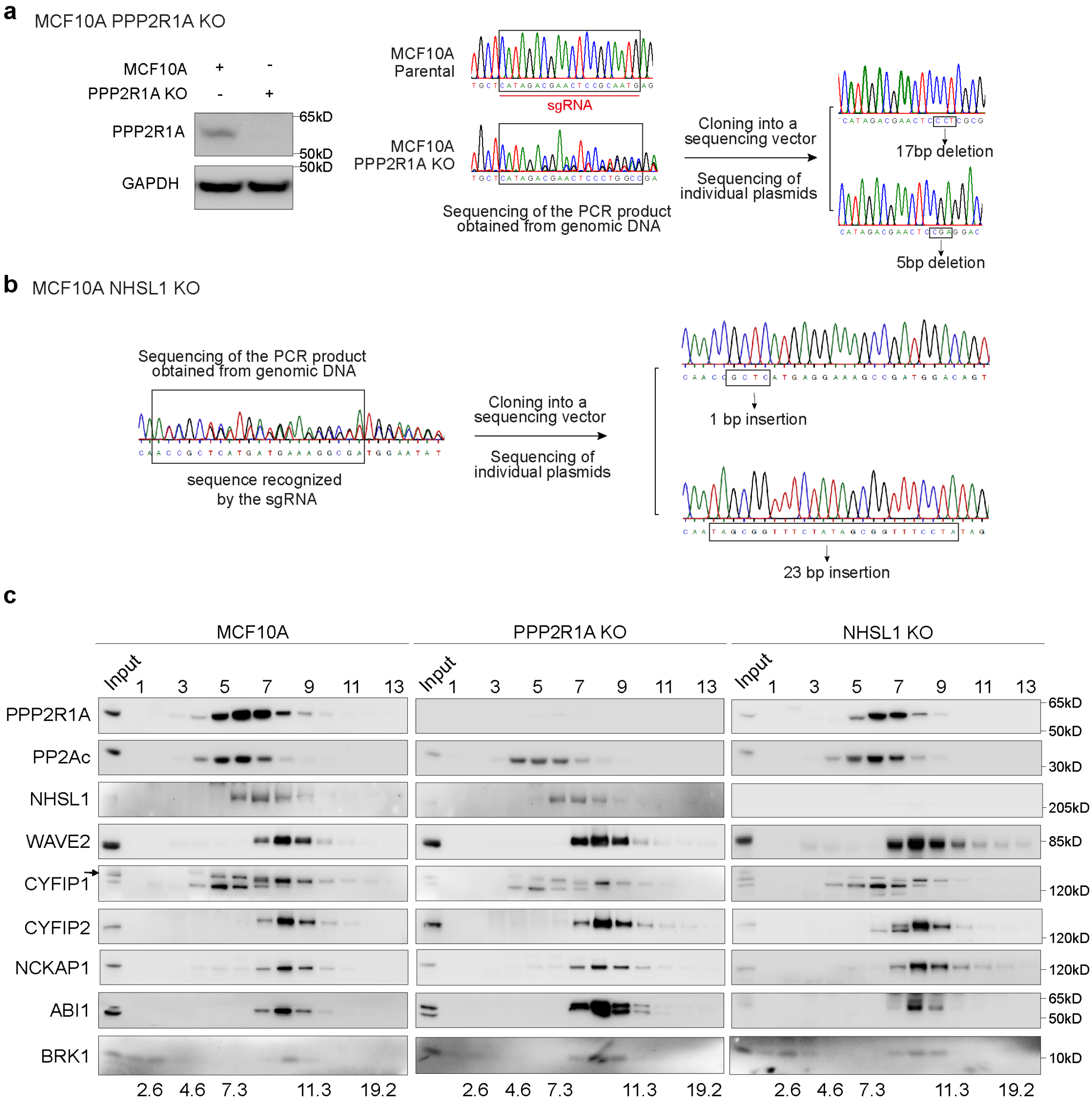

Supplementary Figure 5. Characterization of MCF10A KO cell lines and sedimentation of multiprotein complexes in sucrose gradients. (a) Characterization of the *PPP2R1A* KO clone. Western blots of PPP2R1A and GAPDH as a loading control. Both alleles contain a deletion that induces a frameshift (17 bp and 5 bp). 3 biological repeats with similar results. **(b)** Characterization of the *NHSL1* KO clone derived from MCF10A cells. Both alleles contain an insertion that induces a frameshift (1 bp and 23 bp). **(c)** Cytosolic extracts of MCF10A cell lines were fractionated by ultracentrifugation on 5-20 % sucrose gradients. Elution fractions were revealed by Western blots with the indicated antibodies. Sedimentation coefficient of markers is indicated in Swedbergs below the blots. The distribution of CYFIP1, NCKAP1, ABI1 and BRK1 does not change upon depletion of PPP2R1A or NHSL1 indicating that WSC levels are low compared to WRC levels. 2 biological repeats with similar results.

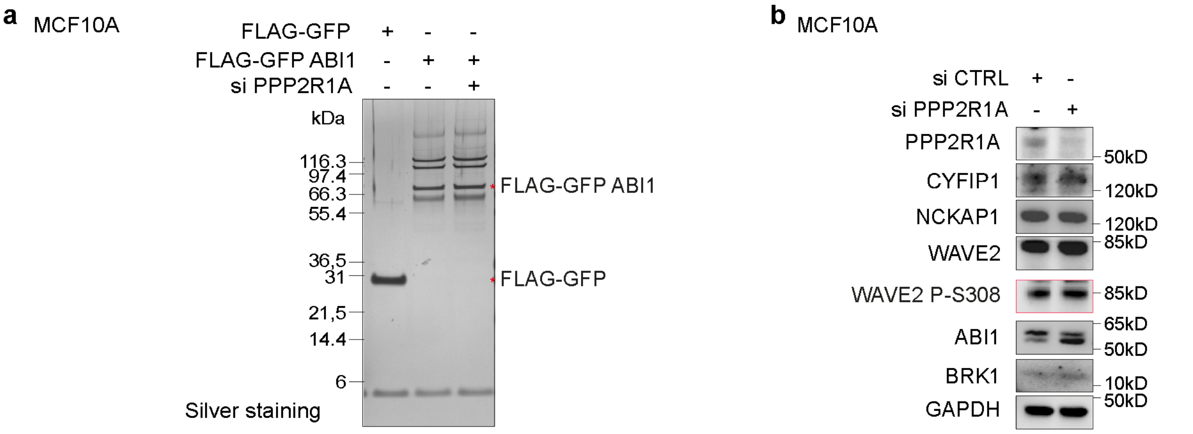

Protein name	Gene name	Number of Phospho (STY)	Position	Probability that the different STY residues are phosphorylated	si CTRL	si PPP2R1A
ABI1	*ABI1*	1	184	TNPPTQKPPS(1)PPMSGR	100 ± 12	73 ± 9
ABI1	*ABI1*	2	188	T(0.004)NPPT(0.015)QKPPS(0.964)PPMS(0.509)GRGT(0.508)LGR	100 ± 9	0
ABI1	*ABI1*	1	201	NT(0.013)PY(0.053)KT(0.934)LEPVKPPTVPNDYMTSPAR	100 ± 49	0
ABI1	*ABI1*	2	192	TNPPTQKPPS(0.998)PPMS(0.244)GRGT(0.758)LGR	100 ± 34	0
NHSL1	*NHSL1*	1	1233	AVPS(0.986)PT(0.011)T(0.003)GEEGSVHSR	100 ± 6	75 ± 9
NHSL1	*NHSL1*	1	328	LDSDAGFHS(1)LPR	100 ± 47	46 ± 0
NHSL1	*NHSL1*	2	1388	NHS(0.984)PS(0.988)PPVT(0.029)PTGAAPSLASPK	100 ± 15	105 ± 0
NHSL1	*NHSL1*	1	1190	SPGAPSAGEAEARPS(0.001)PS(0.002)T(0.002)T(0.002)PLPDS(0.583)S(0.355)PS(0.054)R	100 ± 0	106 ± 45
NHSL1	*NHSL1*	1	209	RKT(0.997)IT(0.003)GVPDNIQK	100 ± 31	64 ± 14
WAVE2	*WASF2*	1	293	RS(0.209)S(0.765)VVS(0.026)PSHPPPAPPLGSPPGPK	100 ± 19	53 ± 12
WAVE2	*WASF2*	1	308	SSVVS(0.004)PS(0.003)HPPPAPPLGS(0.992)PPGPK	100 ± 0	262 ± 97
WAVE3	*WASF3*	1	235	LSQSVYHGAS(0.001)S(0.002)EGS(0.017)LS(0.918)PDT(0.063)R	100 ± 14	57 ± 13

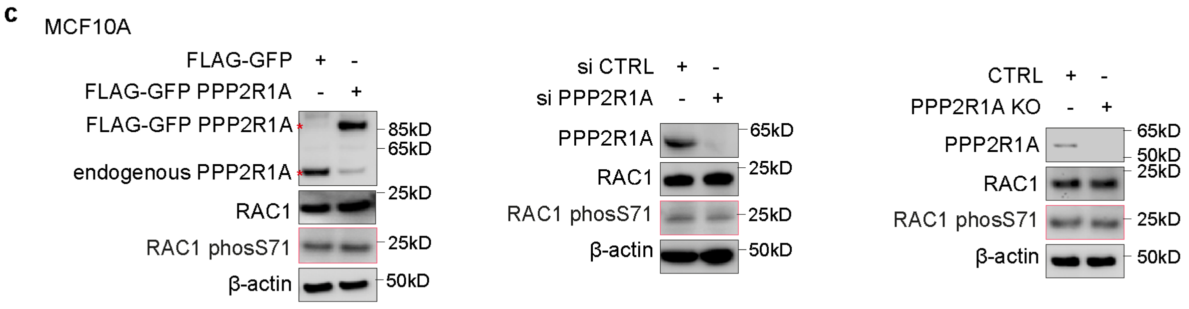

Supplementary Figure 6. Analysis of phosphorylated sites in RAC1, WRC and WSC in presence or absence of PP2R1A. (a) MCF10A stably expressing FLAG-GFP ABI1 were transfected with siRNAs targeting PPP2R1A or control siRNAs. TAP purification of ABI1 was analyzed by SDS-PAGE and by mass spectrometry. Label-free quantification of phosphosites identified by mass spectrometry in subunits of WRC and WSC. Label-free quantification of phosphosites is not as reliable as that of proteins, which are quantified by several peptides. 3 biological repeats, mean ± sem. Only one phosphosite, phosphoserine 308 of WAVE2, highlighted in the table, was found increased when PPP2R1A was depleted. **(b)** Western blots of WRC subunits including phospho-serine 308 of WAVE2. Phosphorylation of serine 308 of WAVE2 is in fact not altered by PPP2R1A depletion. 3 biological repeats with similar results. **(c)** Western blots of RAC1 including phospho-serine 71. The phosphorylation of RAC1 serine 71 is not altered by PPP2R1A depletion or overexpression. 3 biological repeats with similar results.

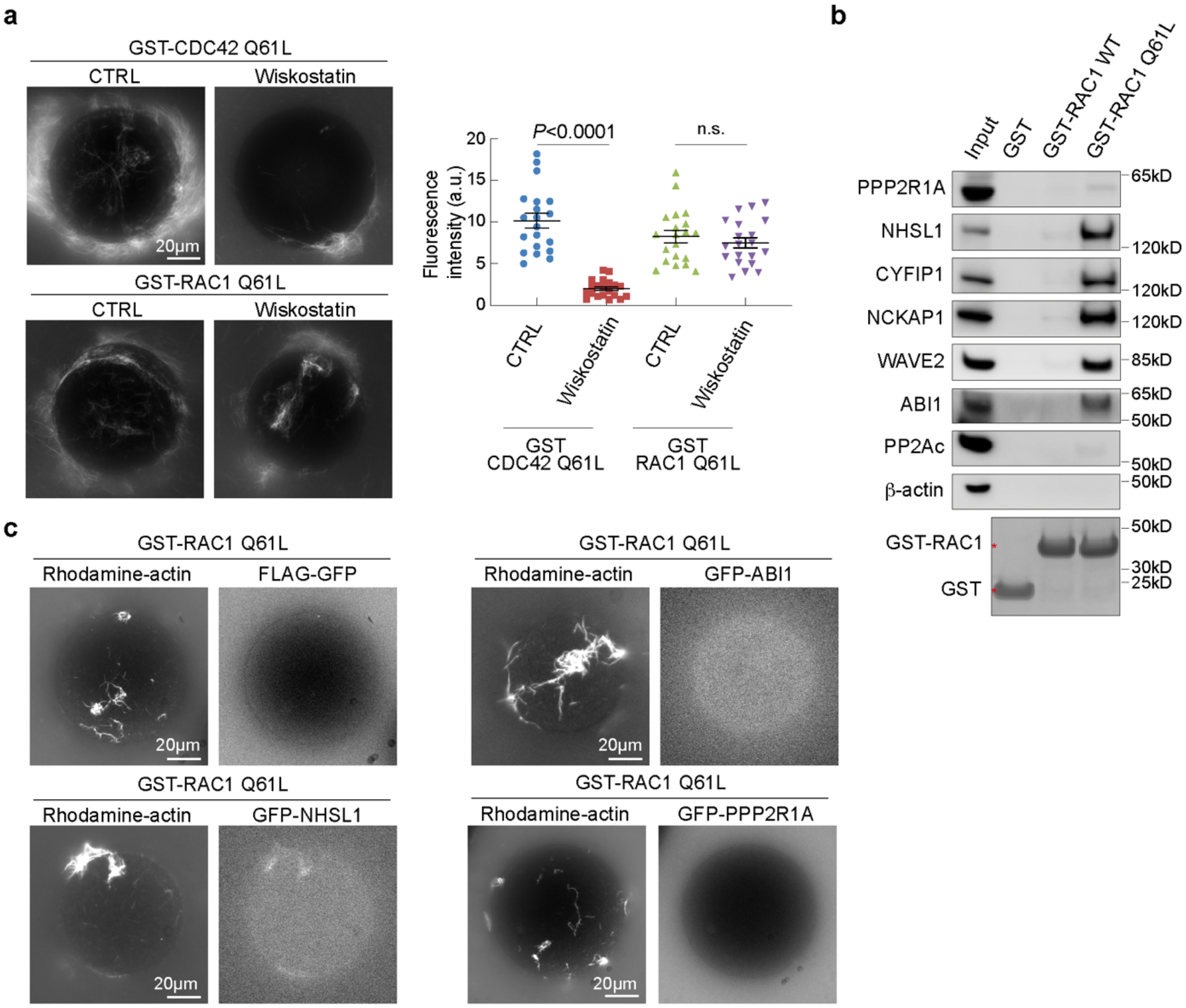

Supplementary Figure 7. Actin polymerization at the surface of beads displaying GST fusions with small GTPases. (a) Beads coated with GST-CDC42 Q61L or GST-RAC1 Q61L were incubated with extracts of MCF10A cells treated or not with 10 µM wiskostatin. Structures containing rhodamine-labeled actin were examined at the surface of beads by epifluorescence and their intensity quantified. n=20. Data are presented as mean ± SEM. n corresponds to the total number of beads quantified in all 3 experiments. Statistical significance was calculated with two-tailed unpaired t-test and P values are indicated. **(b)** Beads coated with GST, GST-RAC1 WT or GST-RAC1 Q61L were incubated with MCF10A cell extracts, then subjected to GST pull down. Western blots with the indicated antibodies. **(c)** Beads coated with GST-RAC1Q61L were incubated with cell extracts from stable MCF10A clones expressing FLAG-GFP tagged ABI1, NHSL1, PPP2R1A or FLAG-GFP as a control. ABI1 and NHSL1 are enriched at the surface of the beads unlike the FLAG-GFP control and PPP2R1A. 3 biological repeats with similar results, only one is displayed. Source data are provided as a Source Data file.

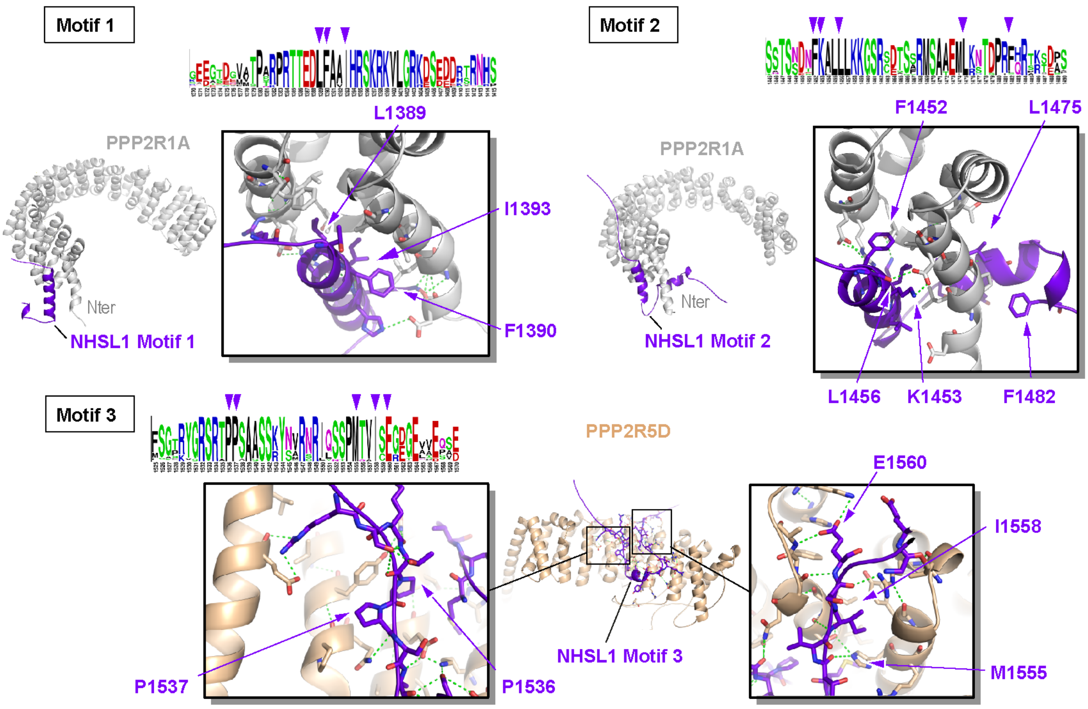

Supplementary Figure 8. Structural motifs of NHSL1 fragment 4. AlphaFold2 predictions of three NHSL1 motifs that interact with PPP2R1A or PPP2R5D subunits of the PP2A complex. Cartoon representation with interacting residues of NHSL1 highlighted as sticks. The conservation pattern represented in a logo plot using WebLogo[1] highlights interacting residues by purple triangles. Displayed complexes are for motif 1, PPP2R1A (grey) and the region P_{1380}SRP-DDH_{1410} in NHSL1 (violet); for motif 2, PPP2R1A (grey) and the region A_{1430}SP-EPS_{1490} in NHSL1 (violet); for motif 3, PPP2R5D (wheat) and the region S_{1522}LS-EPV_{1569} in NHSL1 (violet).

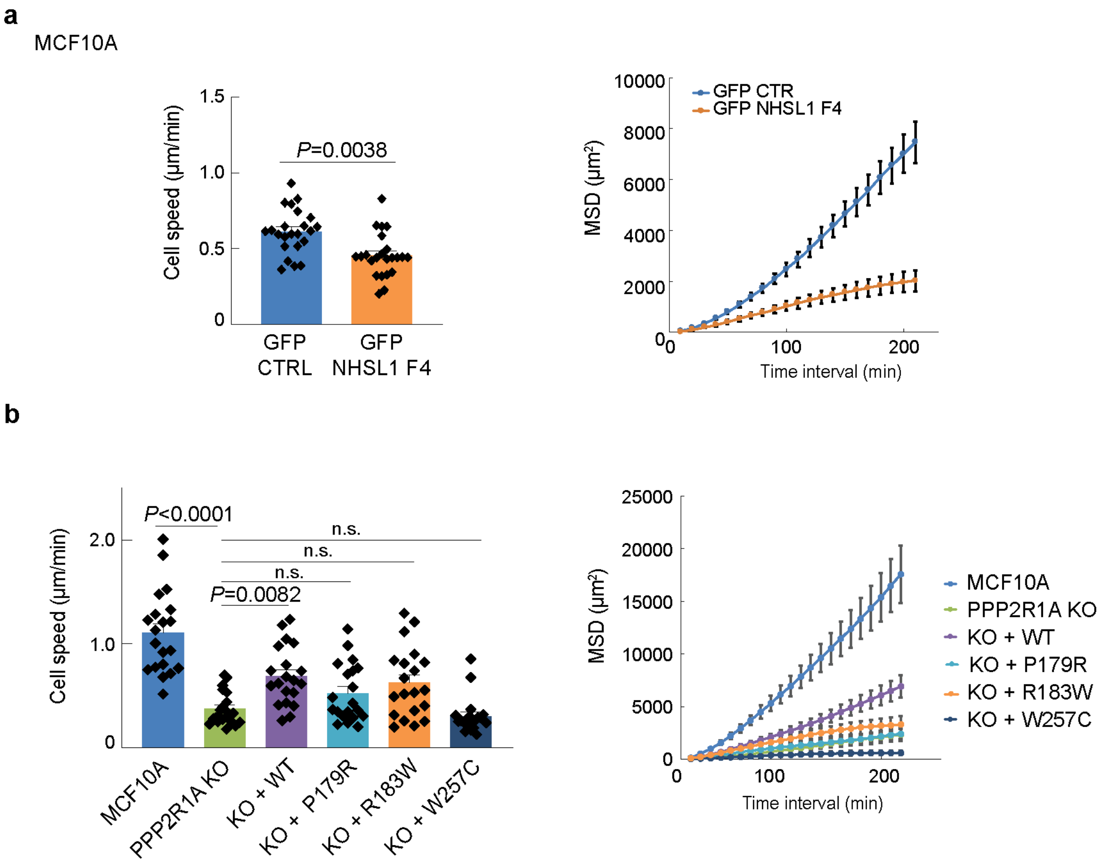

Supplementary Figure 9. Migration parameters of MCF10A cell lines that uncouple PPP2R1A from the WSC. (a) Cell speed and MSD extracted from cell trajectories of MCF10A stably expressing NHSL1 fragment 4, shown in figure 8c. 3 biological repeats of the same experiment with similar results, only one is displayed. Statistical significance was calculated with two-tailed Mann-Whitney test and P values are indicated. **(b)** Cell speed and MSD were extracted from random migration of MCF10A parental cells, *PPP2R1A* knockout cells or KO clones expressing wild type or mutants of PPP2R1A, n=19 cells. Related to the corresponding experiment displayed in figure 9b. Statistical significance was calculated with Kruskal-Wallis test with post hoc Dunn's multiple comparison test and P values are indicated. 3 biological repeats of the same experiment with similar results, only one is displayed. Source data are provided as a Source Data file.

SUPPLEMENTARY METHODS

Mass Spectrometry

The resins containing the immunoprecipitated sample were loaded onto a 10 kDa cutoff centrifugal filters (Microcon, Millipore-Merck) and washed with 500 µL of ammonium bicarbonate buffer (50 mM, pH 8.0, AMBIC). Disulfide reduction was performed adding to the centrifugal filters 200 µL of a solution containing 10 mM dithiothreitol in AMBIC for 2 h at 37°C. Thiol alkylation was performed by adding to the previous samples 200 µL of a solution containing 50 mM iodoacetamide in AMBIC for 30 minutes at room temperature. Reagents were removed by filtration and sample washed three times with 500 µL of AMBIC. Proteins were digested with 1 µg of trypsin/Lys-C (Promega) in 100 µL of AMBIC overnight at 37 °C. The resulting peptide mixture was filtered and acidified with trifluoro acetic acid at a final concentration of 0.1%. Technical triplicates were systematically analyzed.

For each fraction, 6 µL of sample was concentrated on a C18 cartridge (Dionex Acclaim PepMap100, 5 µm, 300 µm i.d. x 5 mm) and eluted on a capillary reverse-phase column (C18 Dionex Acclaim PepMap100, 3 µm, 75 µm i.d. x 50 cm) at 220 nL/min, with a gradient of 2% to 38% of buffer B in 60 min (buffer A: 0.1% aq. Formic Acid/Acetonitrile 98:2 (v/v); buffer B: 0.1% aq. Formic Acid/Acetonitrile 10:90 (v/v)), coupled with a quadrupole-Orbitrap mass spectrometer (Q Exactive HF, ThermoFisher Scientific) using a Top 20 data-dependent acquisition MS experiment: 1 survey MS scan (400-2,000 m/z; resolution 70,000) followed by 20 MS/MS scans on the 20 most intense precursors (dynamic exclusion of 30 s, resolution 17,500).

Protein identification was performed with MaxQuant search engine v.1.5.3.30 against the human Swiss-Prot database (updated in 07/2020), with the following parameter: methionine oxidation, cysteine carbamidomethylation, asparagine/glutamine deamidation and serine/threonine/tyrosine phosphorylation as variable modifications, first search error tolerance 20 ppm, main error tolerance 6 ppm, MS/MS error tolerance 20 ppm, FDR 1%. Quantification was performed in label-free LFQ normalization mode[2] using at least 2 razor or unique peptide per protein. Quantities were estimated using LFQ intensities and normalized by the intensity of the bait protein, ABI1 in the case of Table S1. Proteins found in control samples were filtered out as described in the tables. Significant changes in protein amounts were estimated by ANOVA with Bonferroni's Post-Hoc test using a p-value cutoff of 0.05.

Sucrose gradient

For sucrose gradient analysis of WAVE subunits, Nitrogen cavitation (Parr instruments, 500 Psi for 20 min) followed by centrifugation (16,000 × g, 20 min) and ultracentrifugation (150,000 × g, 60 min) were used to prepare cytosolic extracts from cells trypsinized from two 15 cm dishes and resuspended in the XB buffer (20 mM HEPES, 100 mM NaCl, 1mM MgCl2, 0.1 mM EDTA, 1mM DTT, pH 7.7). 200 µL of extract was loaded on the 11 mL 5–20% sucrose gradient in the XB buffer and subjected to ultracentrifugation for 17 h at 197,000 ×g in the swinging bucket rotor SW41 Ti (Beckman). 0.5 mL fractions were collected and concentrated

by using trichloroacetic acid precipitation with insulin as a carrier. The samples were washed with acetone, dried and then resuspended in the 1x LDS loading buffer with 2.5% of β-ME for Western blot analysis.

Structural Modeling

Sequences of human CYFIP1, NCKAP1, BRK1, ABI2, PPP2R1A and PPP2R5D were retrieved from UniProt database[3] and the full-length NHSL1 cloned in plasmid pCAG were used as input of mmseqs2 homology search program[4] with 3 iterations to generate a multiple sequence alignment (MSA) against the UniRef30 clustered database. Homologs sharing less than 25% sequence identity or less than 50% of coverage of the aligned region with their respective query, were discarded. In case several homologs belonged to the same species, only the one sharing highest sequence identity to the query was kept. Full-length sequences of selected homologs were retrieved and realigned with mafft[5]. To model WSC structure, concatenated MSAs of CYFIP1, NCKAP1, ABI2 (1-160), BRK1 and NHSL1 (1-95, 1-123, or 1-200) were analyzed. Homologs of different subunits belonging to the same species were aligned in a paired manner otherwise in concatenated MSAs. Final concatenated MSAs of WSC contained 2711 positions and 1577 species. MSAs of NHSL1 motif 1 (P_{1380}SRP-DDH_{1410}), motif 2 (A_{1430}SP-EPS_{1490}) were similarly concatenated with that of PPP2R1A and MSA of motif 3 (S_{1522}LS-EPV_{1569}) with that of PPP2R5D (80-530), yielding 3 MSAs from 1733 and 2445 species, respectively. Each concatenated MSA was then used as input to run 5 independent runs of the AlphaFold2 algorithm with 6 iterations each time[6] in order to generate 5 structural models using a local version of the ColabFold interface[7] trained on the multimer dataset[8] on a local HPC equipped with NVIDIA Ampere A100 80Go GPU cards. Best models of each of the 5 runs converged toward similar conformations for each of the 4 modeled molecular systems. High-confidence quality scores of pLDDT in the range of [84.6, 86.2], [91.8, 93.1], [88.3, 88.9], [87.2, 88.9] and pTMscore in the range [0.807, 0.833], [0.816, 0.842], [0.785, 0.8], [0.844, 0.854] were obtained for WSC and the complexes involving NHSL1 motifs 1, 2 and 3, respectively. For each of the four models, the models with highest pTMscores were relaxed using Rosetta relax protocols to remove steric clashes[9] with strong backbone constraints (standard deviation of 0.5 Å for atomic positions) and were used for structural analysis.

SUPPLEMENTARY REFERENCES

1. Crooks, G. E., Hon, G., Chandonia, J.-M. & Brenner, S. E. WebLogo: A Sequence Logo Generator. *Genome Res* 14, 1188–1190 (2004).

2. Cox, J. *et al.* Accurate Proteome-wide Label-free Quantification by Delayed Normalization and Maximal Peptide Ratio Extraction, Termed MaxLFQ*. *Mol Cell Proteomics* 13, 2513–2526 (2014).

3. Consortium, T. U. *et al.* UniProt: the universal protein knowledgebase in 2021. *Nucleic Acids Res* 49, D480–D489 (2020).

4. Steinegger, M. & Söding, J. MMseqs2 enables sensitive protein sequence searching for the analysis of massive data sets. *Nat Biotechnol* 35, 1026–1028 (2017).

5. Katoh, K. & Standley, D. M. MAFFT Multiple Sequence Alignment Software Version 7: Improvements in Performance and Usability. *Mol Biol Evol* 30, 772–780 (2013).

6. Jumper, J. *et al.* Highly accurate protein structure prediction with AlphaFold. *Nature* 596, 583–589 (2021).

7. Mirdita, M. *et al.* ColabFold - Making protein folding accessible to all. *Biorxiv* 2021.08.15.456425 (2022) doi:10.1101/2021.08.15.456425.

8. Evans, R. *et al.* Protein complex prediction with AlphaFold-Multimer. *Biorxiv* 2021.10.04.463034 (2022) doi:10.1101/2021.10.04.463034.

9. Leman, J. K. *et al.* Macromolecular modeling and design in Rosetta: recent methods and frameworks. *Nat Methods* 17, 665–680 (2020).

LEGENDS TO SUPPLEMENTARY TABLES

Supplementary Table 1. ABI1 partners. Label-free quantification of proteins identified by mass spectrometry in TAP purifications of FLAG-GFP ABI1 from parental and RAC1 Q61L expressing MCF10A cells.

Supplementary Table 2. PPP2R1A partners. Label-free quantification of proteins identified by mass spectrometry in TAP purifications of FLAG-GFP PPP2R1A from MCF10A and MDA-MB-231 cells.

Supplementary Table 3. Composition of the WSC. Label-free quantification of proteins identified by mass spectrometry in the TAP purifications of MCF10A cells expressing tagged PPP2R1A and tagged BRK1.

Supplementary Table 4. Phosphosites identified on ABI1 and its partners. Phosphopeptide MS intensity quantified in control or PPP2R1A-depleted MCF10A cells.
