# Supplementary material for: PPP2R1A regulates migration persistence through the NHSL1-containing WAVE Shell Complex
Source: Nat Commun. 2023 Jun 15;14:3541. doi: 10.1038/s41467-023-39276-w (PMC10272187; doi:10.1038/s41467-023-39276-w)
Supplement: Supplementary file 6 — Description to Additional Supplementary Information [file 41467_2023_39276_MOESM6_ESM.pdf]

Description of Additional Supplementary Data Files

Supplementary Movie 1, related to figure 1c. Effect of PPP2R1A depletion on the migration persistence of MCF10A cells. MCF10A cells transfected with pools of control (CTRL) or PPP2R1A siRNAs were recorded and tracked. Scale bar: 40 µm

Supplementary Movie 2, related to figure 1d. Effect of PPP2R1A depletion on the migration persistence of MCF10A RAC1 Q61L cells. MCF10A RAC1 Q61L cells transfected with pools of control (CTRL) or PPP2R1A siRNAs were recorded and tracked. Scale bar: 40 µm

Supplementary Movie 3, related to figure 1e. Effect of PPP2R1A overexpression on the migration persistence of MCF10A cells. MCF10A cells stably expressing FLAG-GFP or FLAG-GFP PPP2R1A were recorded and tracked. Scale bar: 40 µm.

Supplementary Movie 4, related to figure 1f. Effect of PPP2R1A depletion on migration of MDA-MB231 cells in 3D collagen gels. MDA-MB-231 cells transfected with pools of control (CTRL) or PPP2R1A siRNAs were recorded. Scale bar: 40 µm.

Supplementary Movie 5, related to figure 4a. Localization of PPP2R1A and NHSL1 in B16-F1 cells. B16-F1 cells transiently transfected with mScarlet-PPP2R1A and GFPNHSL1 were recorded. Scale bar: 10 µm.

Supplementary Movie 6, related to figure 4c. Localization of PPP2R1A and ARPC1B in B16-F1 cells. B16-F1 cells transiently transfected with mScarlet-PPP2R1A and GFPARPC1B were recorded. Scale bar: 10 µm.

Supplementary Movie 7, related to figure 5a. Dynamics of GFPPPP2R1A localization in B16-F1 cells. B16-F1 cells transiently transfected with GFP-PPP2R1A were subjected to photobleaching (red area) during 1.2 s at time 0. Please note that during photobleaching, only the photobleached area is illuminated. Scale bar: 10 µm.

Supplementary Movie 8, related to figure 5d. Dynamics of GFP-NHLS1 and mScarlet-PPP2R1A localization in B16-F1 cells. B16-F1 cells transiently transfected with GFP-NHSL1 and mScarletPPP2R1A were subjected to photobleaching during 1.2 s at time 0. Scale bar: 10 µm.

Supplementary Movie 9, related to figure 6a. Effect of PPP2R1A and NHSL1 combined depletion on the migration persistence of MCF10A cells. MCF10A cells transfected with indicated siRNA pools were recorded and tracked. Scale bar: 40 µm.

Supplementary Movie 10, related to figure 6b. Effect of PPP2R1A and NHSL1 combined depletion on the haptotaxis of MCF10A cells along the fibronectin gradient. MCF10A cells transfected with indicated siRNA pools were recorded and tracked. Scale bar: 40 µm.

Supplementary Movie 11, related to figure 8c. Effect of NHSL1 fragment 4 on the migration persistence of MCF10A cells. MCF10A cells stably transfected with GFP or GFP-NHSL1 fragment 4 were recorded and tracked. Scale bar: 40 µm.

Supplementary Movie 12, related to figure 9b. Effect of PPP2R1A mutations on the migration persistence of MCF10A cells. PPP2R1A knockout cells were generated in MCF10A. Then the knockout cells were stably transfected with WT or mutant forms of PPP2R1A. Each cell line was recorded and tracked. Scale bar: 40 µm.
