# Supplementary material for: PPP2R1A regulates migration persistence through the NHSL1-containing WAVE Shell Complex
Source: Nat Commun. 2023 Jun 15;14:3541. doi: 10.1038/s41467-023-39276-w (PMC10272187; doi:10.1038/s41467-023-39276-w)
Supplement: Supplementary file 21 — Source Data [file 41467_2023_39276_MOESM21_ESM.zip › Gautreau Source Data Blots.pdf]

Figure1c

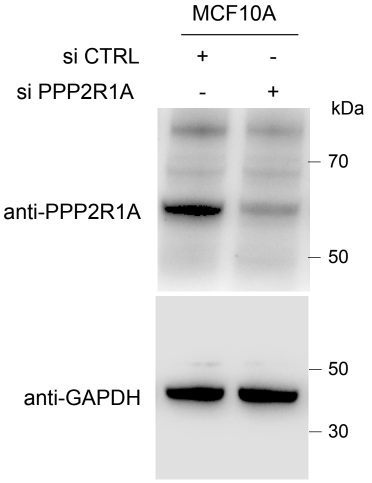

Figure1d

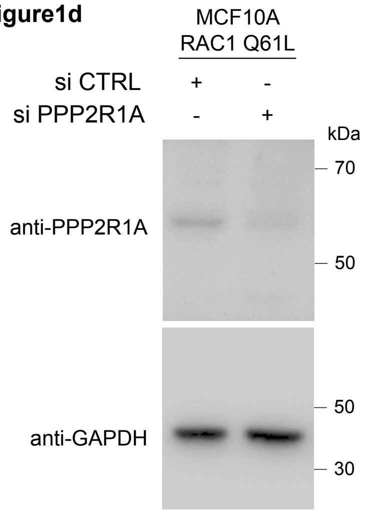

Figure1e

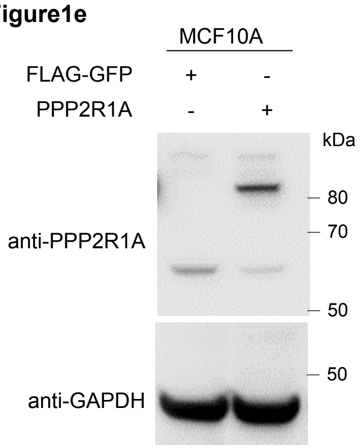

Figure1f

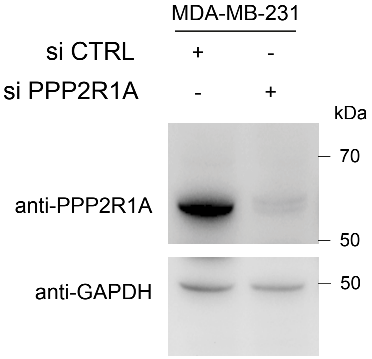

Figure 2c

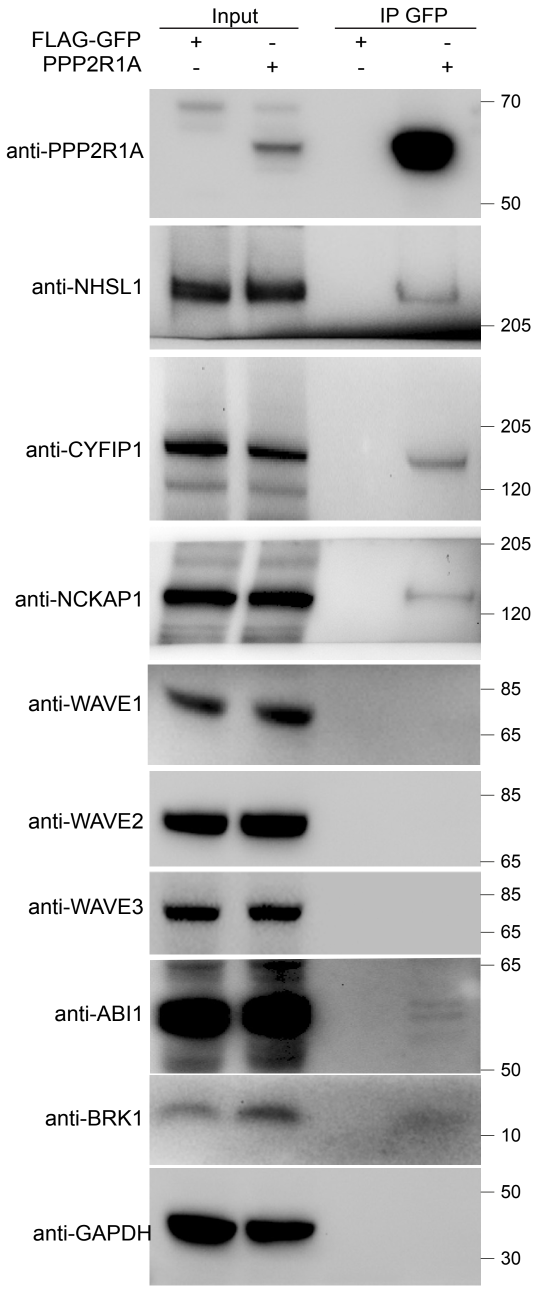

Figure 2d

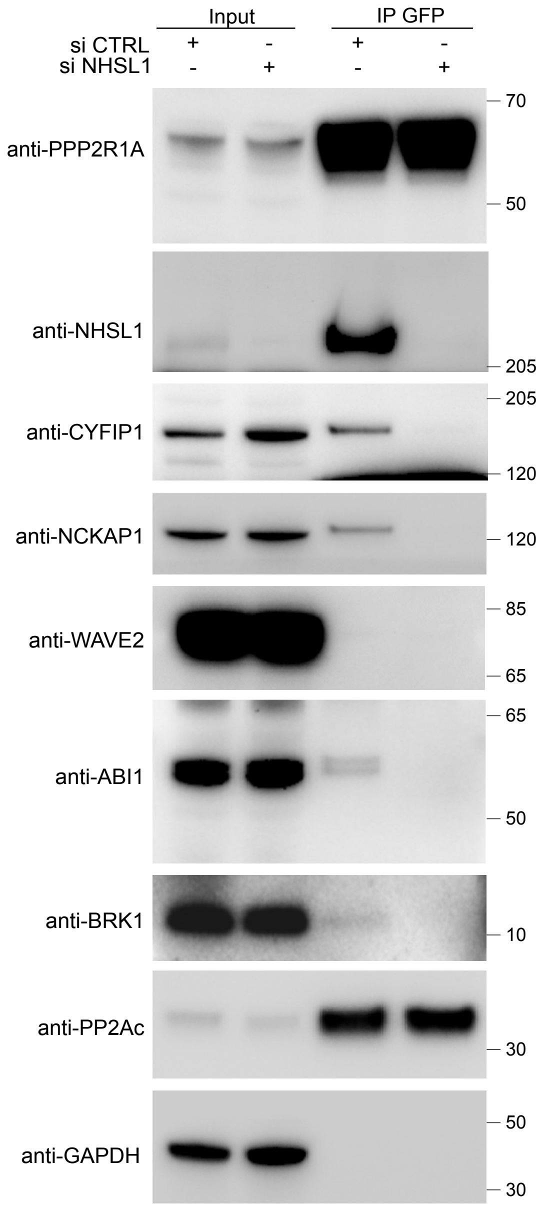

Figure 6a

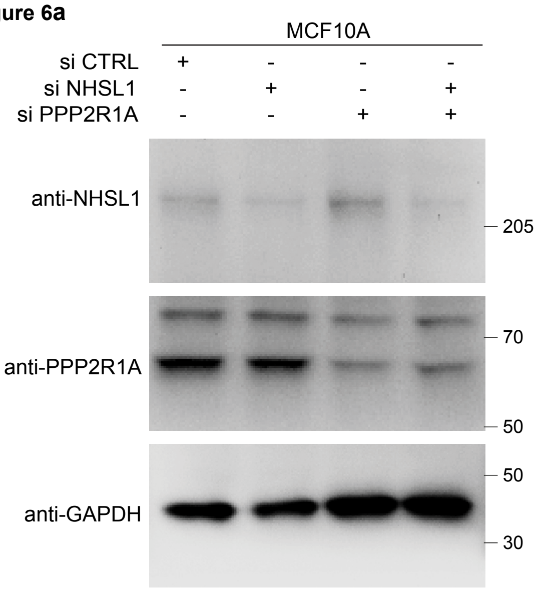

Figure 6b

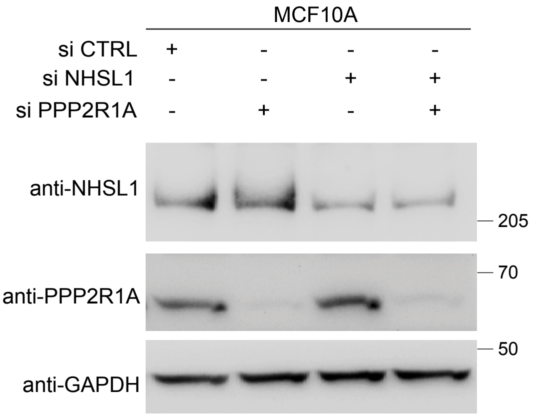

Figure 8a

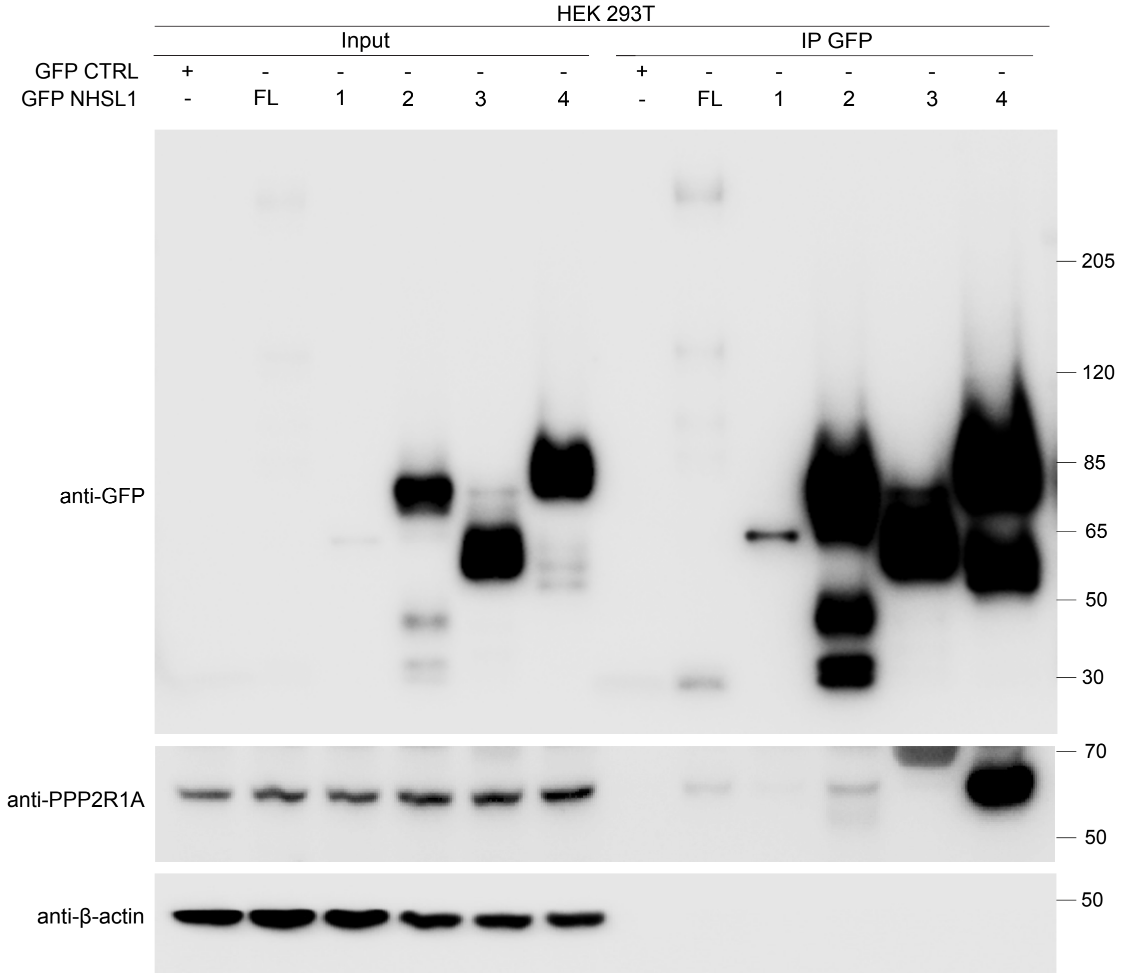

HEK 293T
Input
IP GFP
GFP CTRL
GFP NHSL1
+ - - - - - + - - - - -
- FL 1 2 3 4 - FL 1 2 3 4
anti-GFP
anti-PPP2R1A
anti-β-actin
205
120
85
65
50
30
70
50
50

Figure 9a

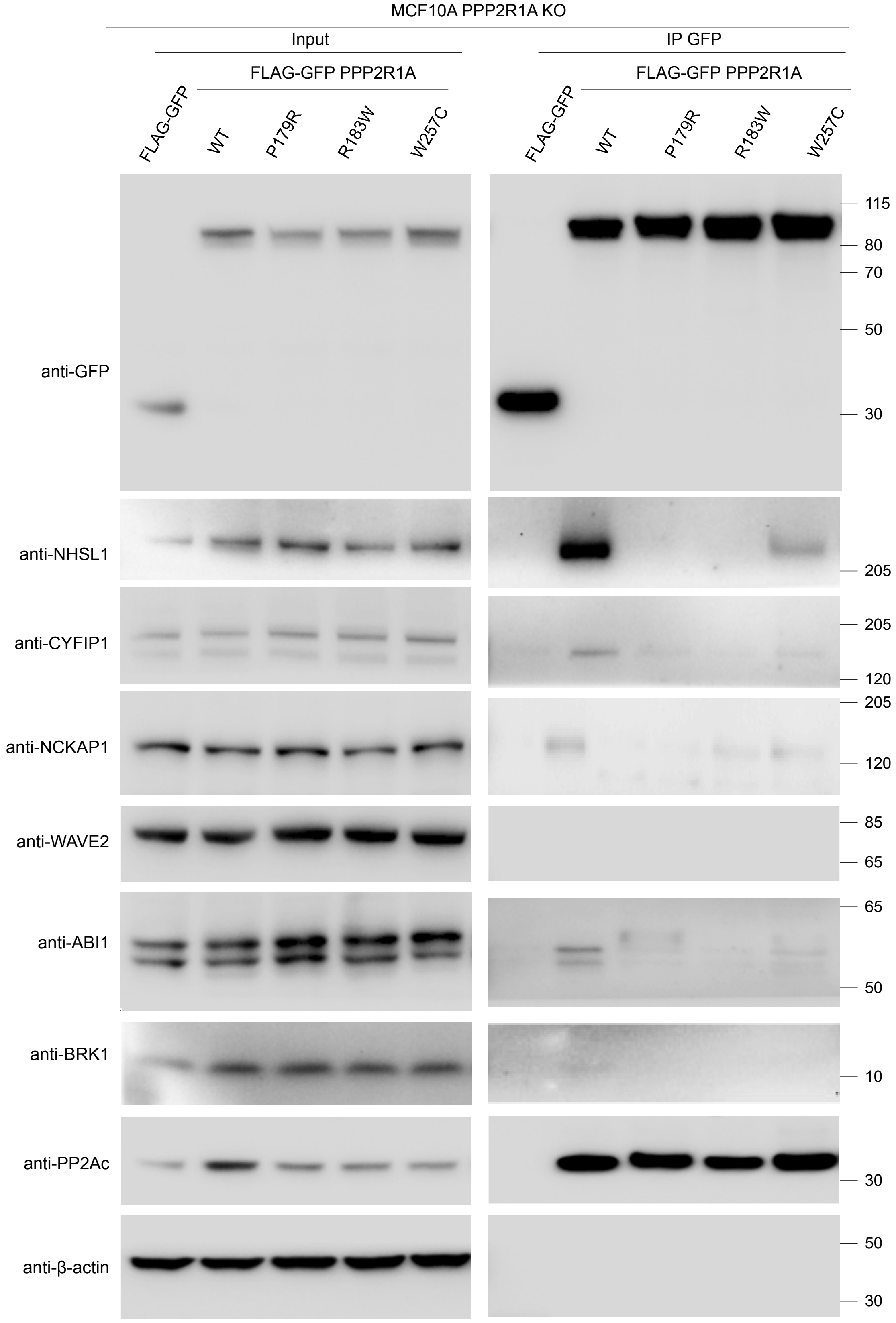
MCF10A PPP2R1A KO
Input
IP GFP
FLAG-GFP PPP2R1A
FLAG-GFP PPP2R1A
FLAG-GFP
WT
P179R
R183W
W257C
FLAG-GFP
WT
P179R
R183W
W257C
anti-GFP
anti-NHSL1
anti-CYFIP1
anti-NCKAP1
anti-WAVE2
anti-ABI1
anti-BRK1
anti-PP2Ac
anti-β-actin
115
80
70
50
30
205
205
120
205
120
85
65
65
50
10
30
50
30

Figure S1a

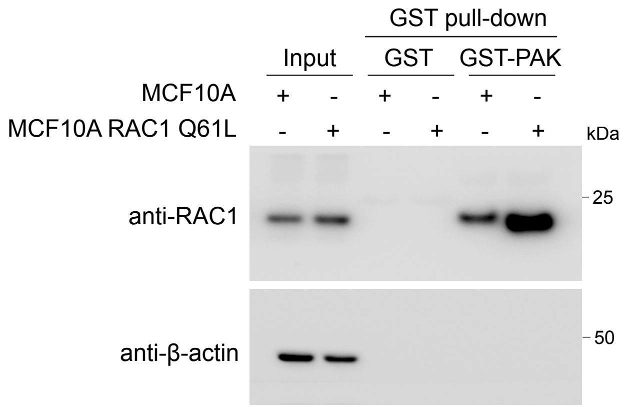

Figure S1d

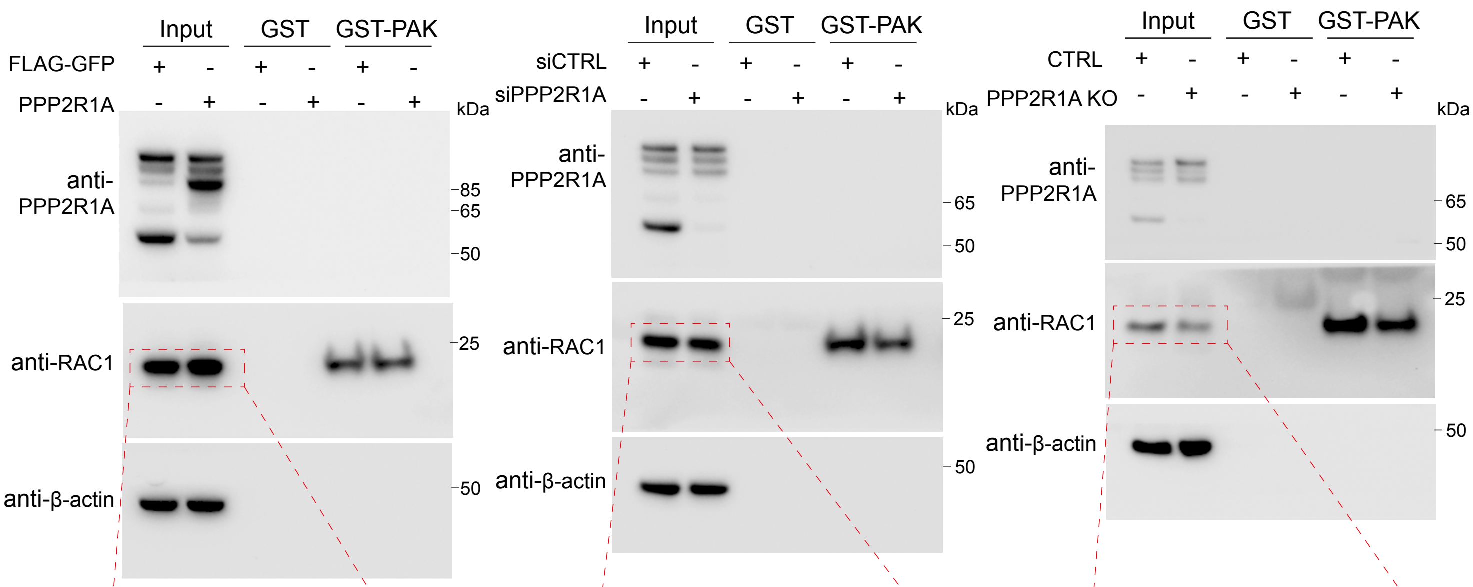

Figure S1e

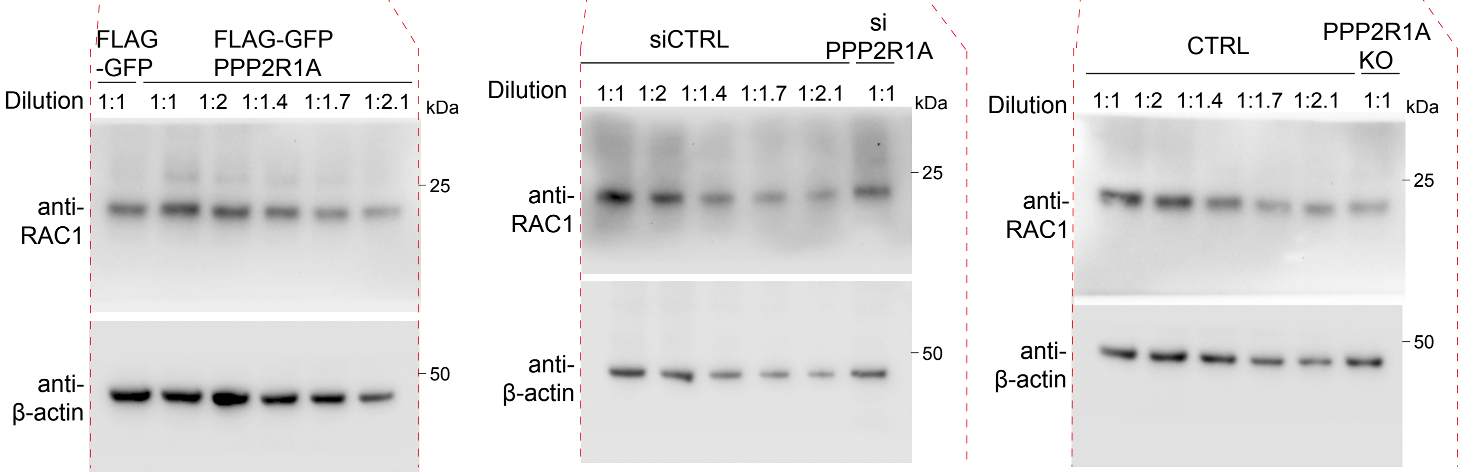

Figure S2e

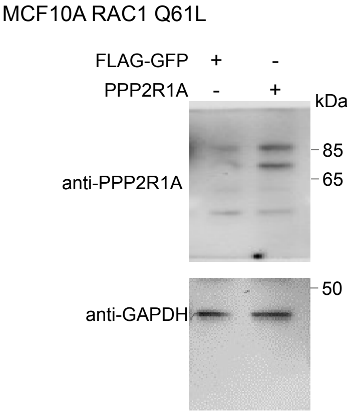

Figure S2f

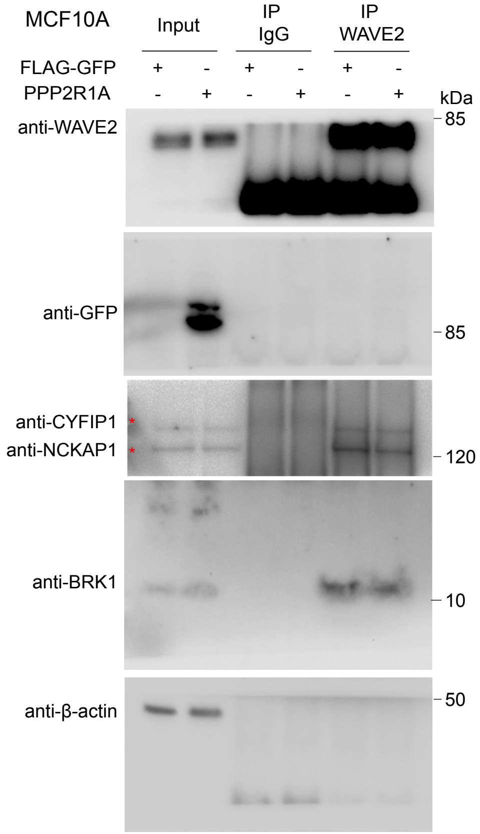

Figure S2g

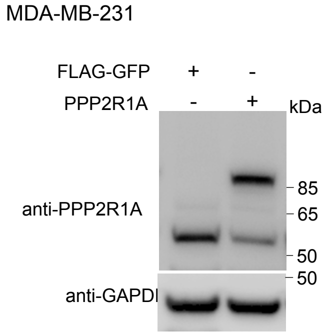

Figure S4a

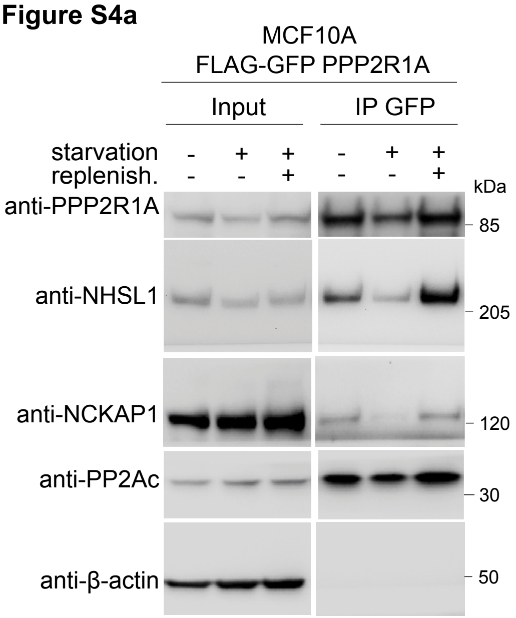

Figure S4c

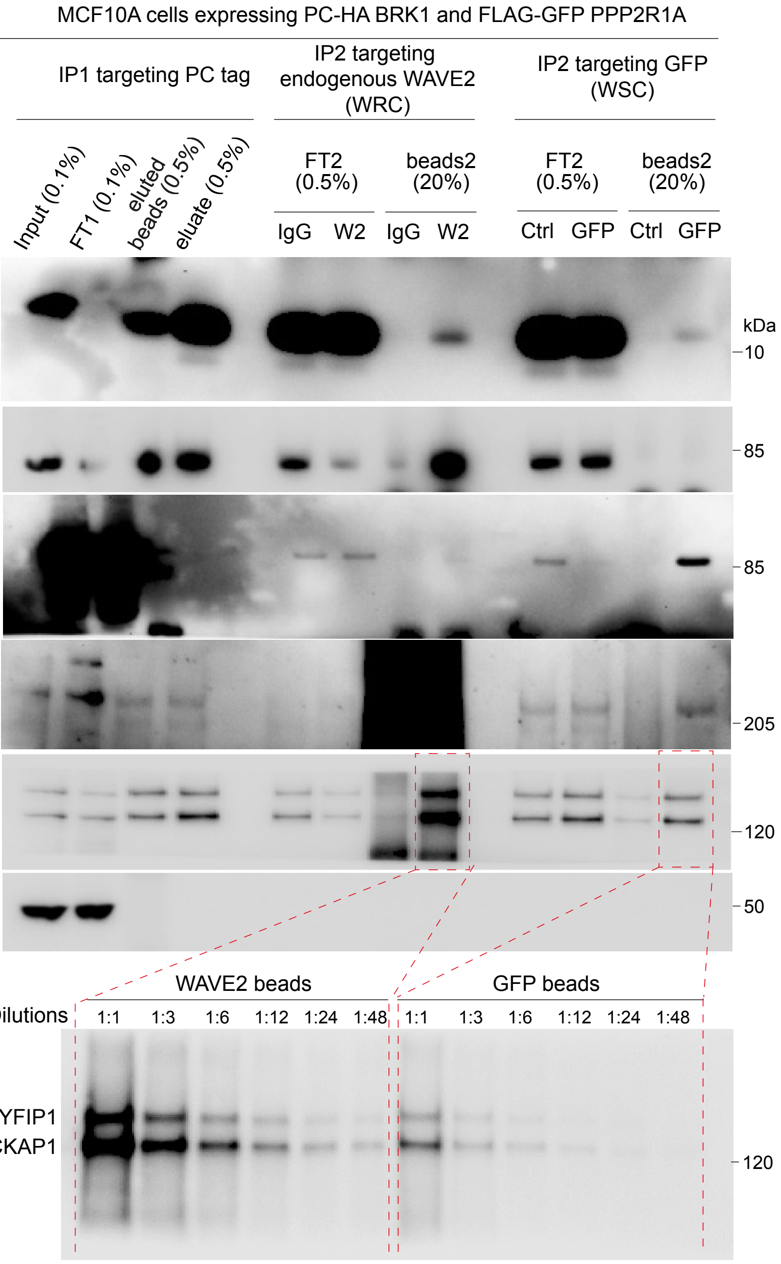

Figure S5a

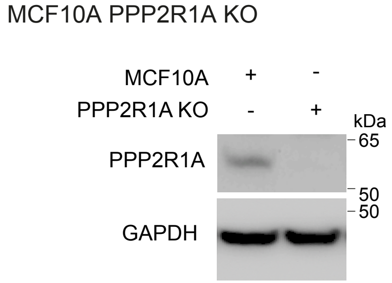

Figure S5c

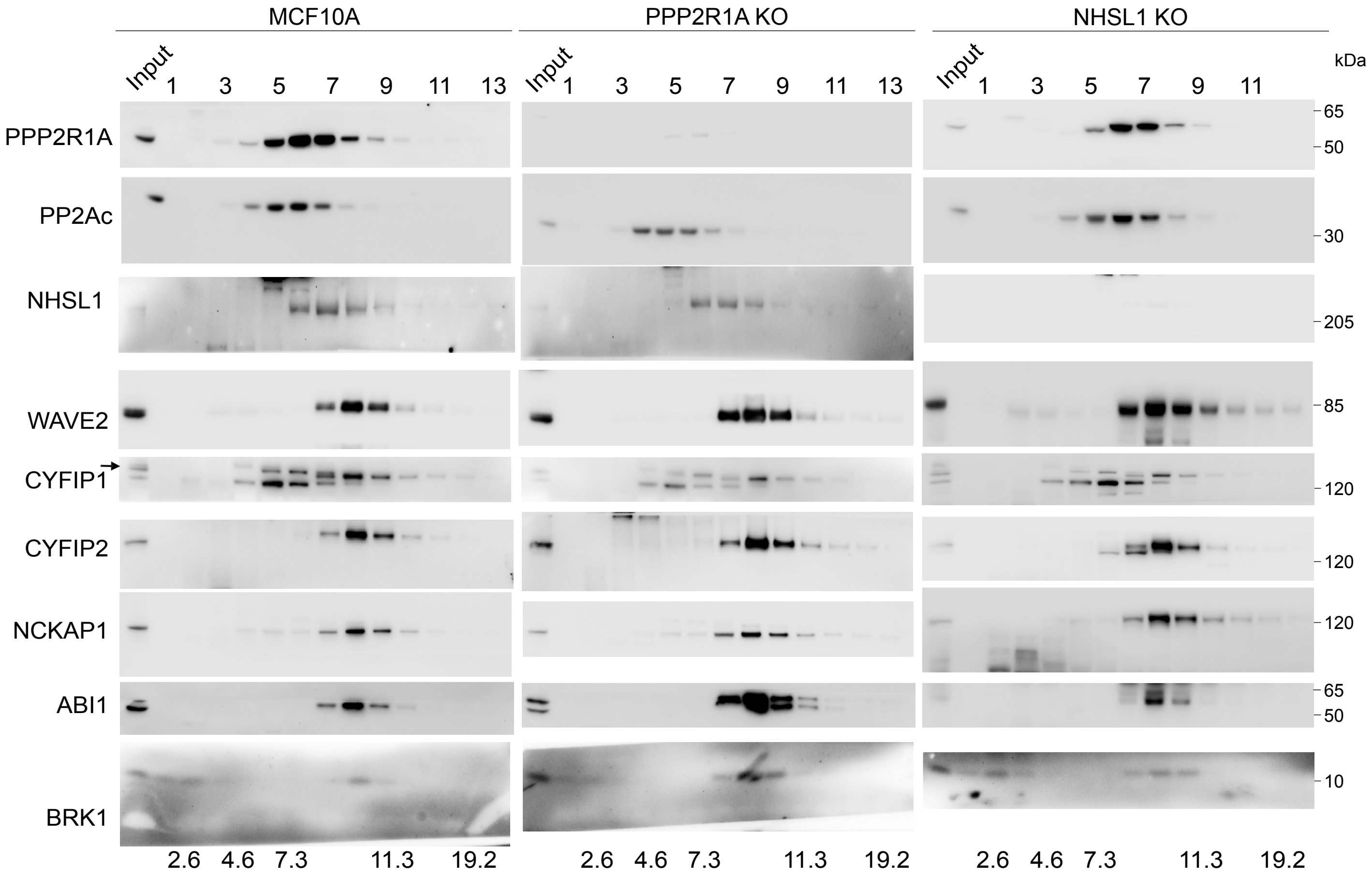

Figure S6b

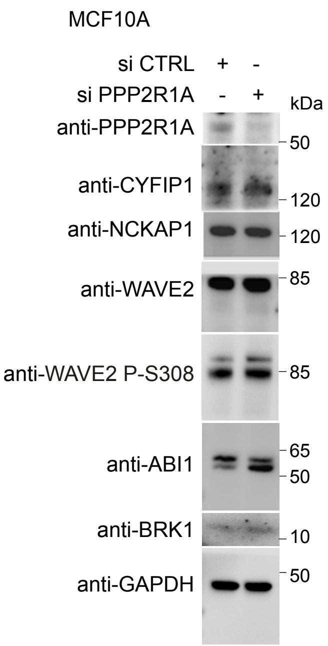

Figure S6c

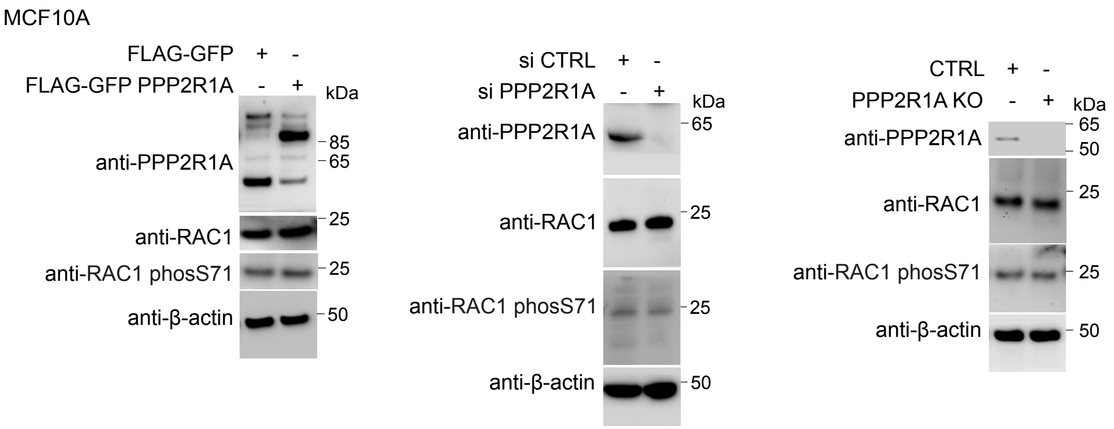

Figure S7b

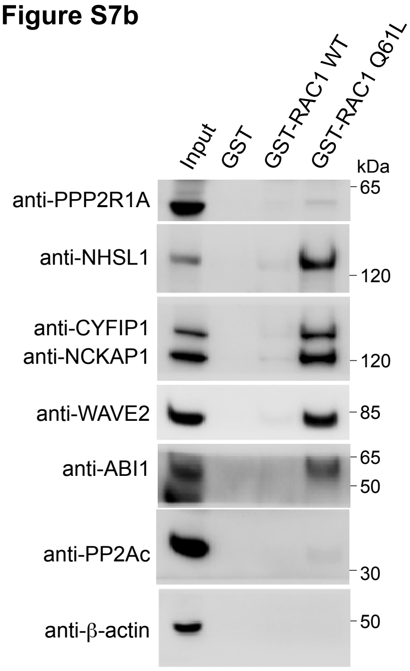
Input
GST
GST-RAC1 WT
GST-RAC1 Q61L
kDa
anti-PPP2R1A
65
anti-NHSL1
120
anti-CYFIP1
anti-NCKAP1
120
anti-WAVE2
85
anti-ABI1
65
50
anti-PP2Ac
30
anti-β-actin
50
